# Photostable and Biocompatible Fluorescent Silicon Nanoparticles for Imaging-Guided Co-Delivery of siRNA and Doxorubicin to Drug-Resistant Cancer Cells

**DOI:** 10.1007/s40820-019-0257-1

**Published:** 2019-03-25

**Authors:** Daoxia Guo, Xiaoyuan Ji, Fei Peng, Yiling Zhong, Binbin Chu, Yuanyuan Su, Yao He

**Affiliations:** Laboratory of Nanoscale Biochemical Analysis, Jiangsu Key Laboratory for Carbon-Based Functional Materials and Devices, Institute of Functional Nano and Soft Materials (FUNSOM), Soochow University, Suzhou, 215123 Jiangsu People’s Republic of China

**Keywords:** Fluorescent silicon nanoparticles, Drug resistance, Gene therapy, Bioimaging

## Abstract

The development of effective and safe vehicles to deliver small interfering RNA (siRNA) and chemotherapeutics remains a major challenge in RNA interference-based combination therapy with chemotherapeutics, which has emerged as a powerful platform to treat drug-resistant cancer cells. Herein, we describe the development of novel all-in-one fluorescent silicon nanoparticles (SiNPs)-based nanomedicine platform for imaging-guided co-delivery of siRNA and doxorubicin (DOX). This approach enhanced therapeutic efficacy in multidrug-resistant breast cancer cells (i.e., MCF-7/ADR cells). Typically, the SiNP-based nanocarriers enhanced the stability of siRNA in a biological environment (i.e., medium or RNase A) and imparted the responsive release behavior of siRNA, resulting in approximately 80% down-regulation of P-glycoprotein expression. Co-delivery of P-glycoprotein siRNA and DOX led to > 35-fold decrease in the half maximal inhibitory concentration of DOX in comparison with free DOX, indicating the pronounced therapeutic efficiency of the resultant nanocomposites for drug-resistant breast cancer cells. The intracellular time-dependent release behaviors of siRNA and DOX were revealed through tracking the strong and stable fluorescence of SiNPs. These data provide valuable information for designing effective RNA interference-based co-delivery carriers.

**Electronic supplementary material:**

The online version of this article (10.1007/s40820-019-0257-1) contains supplementary material, which is available to authorized users.

## Highlights

- A novel all-in-one fluorescent nanomedicine platform based on silicon nanoparticles (SiNPs) was developed for imaging-guided co-delivery of short interfering RNA (siRNA) and doxorubicin (DOX).
- The intracellular time-dependent release behaviors of siRNA and DOX were visually monitored by tracking the strong and stable fluorescence of SiNPs.
- The SiNPs-based nanocarriers displayed pronounced therapeutic efficiency on drug-resistant breast cancer cells.

**Electronic supplementary material:**

The online version of this article (10.1007/s40820-019-0257-1) contains supplementary material, which is available to authorized users.

## Graphical abstract

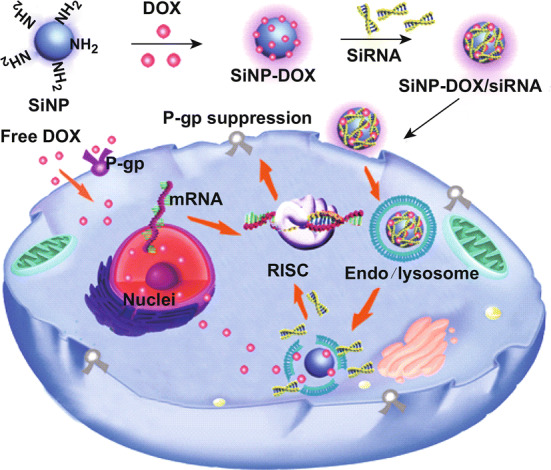

## Introduction

Despite great progress in cancer treatment, multidrug resistance (MDR), which can lead to high recurrence rates and treatment failures, remains a tremendous challenge in cancer chemotherapy [1]. Generally, the abnormal expression of related genes on drug efflux, metabolism and targets, or survival/death signaling pathways accounts for the genetic basis of drug resistance [1–3]. For example, multidrug resistance protein 1 (MDR 1), also known as P-glycoprotein (P-gp) and ABCB1, is a member of the ATP-binding cassette (ABC) transporter protein family, which is overexpressed in many types of cancer cells [2]. Crucially, the overexpression of P-gp promotes the efflux of various hydrophobic chemotherapeutics from cancer cells. This results in an extremely low accumulation of therapeutic agents inside cells and reduces chemotherapeutic efficiency. Since the discovery of RNA interference (RNAi) in *Caenorhabditis elegans* and mammalian cells, synthetic small interfering RNA (siRNA) consisting of approximately 21 to 23-base-pair double-stranded RNA has emerged as a potential therapeutic agent for treatment of various diseases, including cancers [4–7]. The ability of siRNA to specifically and efficiently silence nearly any target gene of interest could be valuable in suppressing the expression of MDR-related proteins, such as P-gp. The combination of chemotherapeutics and siRNA has been recognized as an attractive option for overcoming drug resistance [3, 8, 9].

In seeking to realize such a combination therapy, a critical challenge is the development of effective and safe vehicles to deliver chemotherapeutics and siRNA [10]. Nanomaterials have been extensively explored as an RNAi-based delivery platform to treat cancer cells because of their ability to circumvent drug resistance mechanisms and protect siRNA from biodegradation [11–19]. Nowadays, to further optimize the RNAi nanotherapeutic approach and shorten drug development time, it is important to incorporate imaging modalities into therapy [20–22]. By visualizing, characterizing, and quantifying the biological process (e.g., cellular uptake, subcellular dissociation, and stability) of genes and drugs, the therapeutic effects can be monitored in real time. However, organic dyes (e.g., carboxyfluorescein (FAM) and Alexa Fluor 647) suffer from severe photobleaching, while heavy metal-containing quantum dots might pose potential safety hazards [13, 16]. Consequently, novel, fluorescent, all-in-one nanocarriers with superior optical properties (e.g., high and stable fluorescence) and excellent biocompatibility are needed to facilitate the development of imaging-guided RNAi-based combination therapy in drug-resistant cancer cells.

Recently, fluorescent silicon nanoparticles (SiNPs) that are extremely photostable and possess relatively strong fluorescence have emerged as a novel and promising fluorescent bioprobe in a wide range of optical applications [23–25]. For example, by modifying SiNPs with targeted peptides, the resulting SiNPs bioprobe featuring strong/stable fluorescence (photoluminescence quantum yield, PLQY, of approximately ~ 28%) and small size (< 10 nm) were applied for real-time and long-term imaging of cancer cells [26]. Biosensors based on fluorescent SiNPs with negligible cytotoxicity were developed for the specific and sensitive detection of lysosomal pH fluctuation by the conjugation of pH-sensitive compounds (i.e., dopamine) to SiNPs [27]. In particular, one of our recent studies demonstrated that doxorubicin (DOX) can be loaded on SiNPs to produce SiNPs-based nanocarriers with pronounced fluorescence and robust photostability [28]. The prepared SiNPs-based nanocarriers with adjustable drug-loading capacity were very suitable for optical imaging-guided cancer therapy because of their high fluorescence and robust photostability. However, it remains unknown whether these nanocarriers are available for imaging-guided co-delivery of siRNA and chemotherapeutic agents, facilitating the enhancement of the therapeutic efficacy in MDR cancer cells.

Herein, the fluorescent SiNPs-based nanocarriers were used for MDR cancer cells via the co-delivery siRNAs and DOX. The strong and stable fluorescence signals of SiNPs allowed the long-term fluorescence tracking of the intracellular transport of siRNA and DOX and revealed their time-dependent and dual-responsive release behaviors. Remarkably, the successful MDR1 gene silencing (approximately 80%) by dissociated siRNA enhanced the accumulation of DOX molecules in drug-resistant MCF-7 cells (MCF-7/ADR), which decreased the half maximal inhibitory concentration (IC_50_) of DOX by over 35-fold. Our results suggest that SiNPs-based fluorescent nanocomposites can be used as imaging-guided RNAi-based co-delivery nanoagents for the treatment of MDR cancer cells.

## Experimental

### Preparation and Characterization of SiNP-DOX/siRNA Nanocomposites

Fluorescent SiNPs were synthesized through a photochemical method as described in our previous work [29]. In short, the precursor solution was first prepared by adding 100 mL (3-aminoprophyl) trimethoxysilane (APTES, 97%; SigmaAldrich, USA) containing 20 g 1,8-naphthalimide (SigmaAldrich) to 900 mL Milli-Q water. After a thorough 10-min stirring, the mixture was allowed to react for 40 min at room temperature by exposure to ultraviolet light at 365 nm (Spectroline, USA). To purify the as-prepared SiNPs, the solution was carefully dialyzed in de-ionized water in dialysis bags with a molecular weight cutoff of 1 kDa (Biotopped Life Sciences, China). Thereafter, DOX molecules (Huafeng United Technology Co. Ltd., China) were loaded onto purified SiNPs to prepare SiNP-DOX conjugates as we previously detailed [28]. Excess DOX molecules were removed by ultrafiltration using 10 kDa Nanosep centrifugal devices (Pall Life Sciences, USA). SiNP-DOX/siRNA nanocomposites were further prepared by mixing the resultant SiNP-DOX with 176 ng of P-gp siRNA (GenePharma Co. Ltd., China) (Table S1) with vigorous stirring at different SiNPs/siRNA (w/w) ratios of 30, 90, 150, 210, and 270. The resultant mixtures were analyzed by 1.5% agarose gel electrophoresis. To visualize siRNA patterns, 1% Gel Red (Biotium, USA) was added in the gel. The siRNA patterns were imaged by Imager 600 (Amersham, UK) and quantified by Image J software (NIH, USA) [30].

Transmission electronic microscopy (TEM), high-resolution TEM (HRTEM, CM 200 electron microscope; Philips, USA), dynamic light scattering (DLS, ZEN3690; Malvern Corp, U.K.), ultraviolet–visible (UV–Vis)–near-infrared (NIR) absorption (Lambda 750 spectrophotometer; PerkinElmer, USA) and photoluminescence (PL, FLUOROMAX-4 spectrofluorimeter; HORIBA Jobin Yvon SAS, France) were utilized to characterize the resultant SiNPs, SiNPs-DOX, and SiNP-DOX/siRNA nanocomposites.

### Stability Evaluation

To test the stability of SiNP-DOX/siRNA in the culture media, the as-prepared SiNP-DOX/siRNA nanocomposites (SiNPs/siRNA (w/w) ratio of 210) were first incubated in RPMI-1640 medium for 24 and 48 h at 37 °C. Before the samples were analyzed by 1.5% agarose gel electrophoresis, the siRNA was released from the nanocomposites using 1% heparin. For the RNase A protection assay, SiNP-DOX/siRNA nanocomposites were incubated with 1 ng RNase A at 37 °C for 1 h. The nuclease activity of RNase A was terminated by treatment with 25 mM sodium dodecyl sulfate (SDS, 99%; J&K Scientific Ltd., China) at 60 °C for 5 min. The same analyses as described in Sect. 2.2 were performed on the samples.

### Dual-Responsive Release Behavior

To investigate the release of siRNA, SiNP-DOX/siRNA nanocomposites were incubated in phosphate-buffered saline (PBS) containing high concentrations of phosphate groups (5–40 mM) for 12 h at 37 °C. Next, the samples were analyzed by agarose gel electrophoresis. The release of DOX was further studied by incubating SiNP-DOX/siRNA nanocomposites at pH 5.0, 7.4, and 8.4 for 3, 6, 12, 24, 36, 48, 60, and 72 h. After each treatment, the samples were subjected to ultrafiltration to collect the released DOX, which was quantified using the determined UV–Vis–NIR absorbance spectra. To assess the effect of encapsulated siRNA on DOX release, SiNP-DOX/siRNA nanocomposites pretreated by 1% heparin were set as the control group. SiNP-DOX/siRNA (containing heparin) was then treated as described above.

### Intracellular Distribution

MCF-7 and drug-resistant MCF-7/ADR cells were cultured in RPMI-1640 medium supplemented with 10% (v/v) heat-inactivated fetal bovine serum (FBS) and 1% (v/v) penicillin–streptomycin antibiotics. MCF-7/ADR cells were cultured in wells of 24-well plates on cover slips at a density of 1.2 × 10^5^ cells/well for 24 h and then incubated with nanocomposites for 3, 6, 12, and 24 h. To determine the intracellular localization of SiNP-DOX/siRNA nanocomposites, cells were stained with LysoTracker Green DND-26 (125 nM; Invitrogen, USA) for 40 min. Afterward, the samples were mounted on microscope slides using Fluoromount (F4680; SigmaAldrich), examined by laser scanning confocal microscopy (LSCM) using a model TCS-SP5 microscope (Leica, Germany), and quantified with LAS AF Lite software (Leica). SiNPs and LysoTracker Green DND-26 were excited by 405 and 476 nm; corresponding emission windows were 420 to 480 nm and 500 to 550 nm, respectively.

### Intracellular Release

To study the intracellular release of siRNA and DOX from SiNP-DOX/siRNA nanocomposites, FAM-labeled siRNA (siRNA^FAM^, purchased from GenePharma Co. Ltd., China) was used to fabricate SiNP-DOX/siRNA^FAM^ nanocomposites. MCF-7/ADR cells were incubated with SiNP-DOX/siRNA^FAM^ nanocomposites (A_SiNP_ = 1, DOX = 5 μg mL^−1^, and 100 nM siRNA^FAM^) for 3, 6, and 12 h. After treatment, cells were examined using LSCM. The excitation wavelengths for SiNPs, siRNA^FAM^, and DOX were 405, 476, and 488 nm, and the emission windows were 420 to 480 nm, 500 to 550 nm, and 560 to 650 nm, respectively. All images were captured using the same instrument settings.

### In vitro Gene-Silencing Efficiency

To evaluate the gene-silencing efficiency of SiNP-DOX/siRNA nanocomposites in vitro, quantitative real-time transcription polymerase chain reaction (qRT-PCR) was first employed to quantify intracellular P-gp expression at the mRNA level. MCF-7-/ADR cells were seeded in wells of 24-well plates at a density of 1.5 × 10^5^ cells/well overnight, and then treated with SiNPs, SiNP-DOX, SiNP-DOX/NC siRNA (scrambled siRNA), or SiNP-DOX/siRNA (three strands of P-gp siRNA). After a 24 h incubation, cells were cultured in fresh medium and allowed to grow for another 24 h. After that, the total RNA in each sample was collected according to the established Trizol reagent protocol (Invitrogen, USA) [31] and corresponding cDNA was obtained using PrimeScript^®^ RT reagent kit (Takara Biotechnology Co. Ltd, Japan). The mixture of cDNA, forward and reverse primers (designed by Primer Bank, Table S2) and the SYBR Green Master Mix (Biotool, USA) was run on the CFX96 Real-Time PCR Detection System (Bio-Rad, USA). β-actin was used as an internal loading control (Table S2).

Immunofluorescent staining was utilized to visually evaluate P-gp expression at the protein level. Briefly, MCF-7/ADR cells were treated as described above, fixed with 4% paraformaldehyde containing 4% sucrose for 20 min, and blocked with 4% BSA containing 0.1% Triton X-100 for 40 min. The cells were incubated with P-gp primary mouse monoclonal antibody (1:300, Santa Cruz Biotechnology, USA) for 2 h, followed by washing three times with PBS containing 0.1% Tween 20. Fluorescein isothiocyanate (FITC)-labeled secondary goat anti-mouse antibody (1:300, Santa Cruz Biotechnology) and Hoechst 33258 (3 μg mL^−1^; Beyotime Biotechnology, China) were used to stain cells. Finally, the samples were imaged by LSCM. For Hoechst 33258 staining, the excitation wavelength was 405 nm and the emission window was 420 to 480 nm. The excitation wavelength and corresponding emission window for FITC were 488 nm and 500 to 550 nm.

### MTT Assay

A standard 3-(4,5-dimethylthiazol-2-yl)-2,5-diphenyl tetrazolium bromide (MTT, SigmaAldrich) assay was carried out to evaluate the in vitro therapeutic effect. Cells were seeded in wells of a 96-well plate at a density of 0.8 × 10^4^ cells/well. Different concentrations of free DOX (0.63–10 μg mL^−1^), pure SiNPs (A_SiNP_: 0.125–2), SiNP-DOX (DOX: 0.6310 μg mL^−1^, A_SiNP_: 0.125–2) conjugates, SiNP-DOX/NC siRNA and SiNP-DOX/siRNA (DOX: 0.63–10 μg mL^−1^, A_SiNP_: 0.125–2, siRNA: 12.5–200 nM) nanocomposites were used to treat the cells. After a 72-h incubation, cells were treated with MTT (20 μL, 5 mg mL^−1^) for 4 h and then lysed by 10% acidified SDS. To determine the cell viability, absorbance at 570 nm of each well was determined using the model 680 microplate reader (Bio-Rad). Three independent assays were performed in triplicate for all measurements. SPSS Statistics 17.0 software (SPSS Inc., USA) was used to calculate IC_50_ values.

## Results and Discussion

### Fabrication and Characterization of SiNP-DOX/siRNA Nanocomposites

**Fig. 1:**
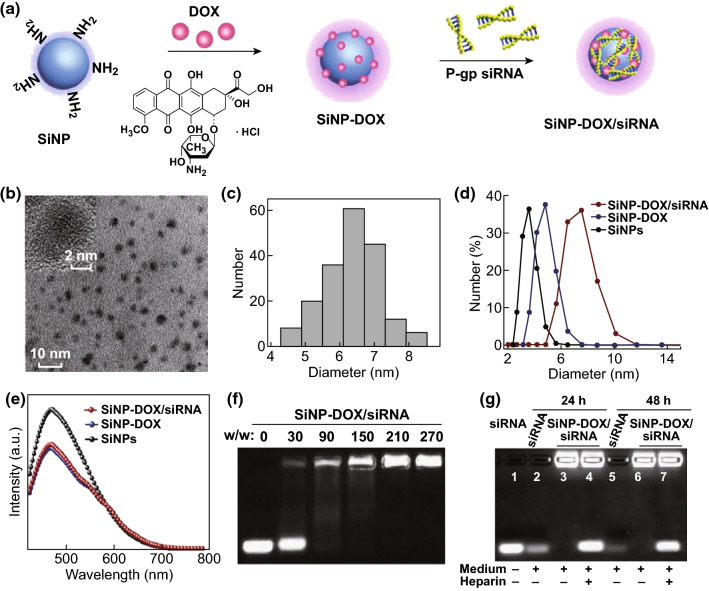
**a** Proposed fabrication scheme of SiNP-DOX/siRNA nanocomposites. **b** TEM image of the prepared SiNP-DOX/siRNA nanocomposites. Insets present the enlarged HRTEM image. **c** Diameter distribution of SiNP-DOX/siRNA nanocomposites determined from TEM. **d** DLS of pure SiNPs (black line), SiNP-DOX conjugates (blue line) and SiNP-DOX/siRNA nanocomposites (red line). **e** Photoluminescence spectra of pure SiNPs (black line), SiNP-DOX conjugates (blue line) and SiNP-DOX/siRNA nanocomposites (red line) upon excitation at 405 nm. **f** Gel retardation electrophoresis of free siRNA and SiNP-DOX/siRNA nanocomposites prepared at different SiNPs/siRNA (w/w) ratios. **g** The stability of SiNP-DOX/siRNA nanocomposites in culture medium (RPMI-1640). Lane 1 is the naked siRNA incubated in DEPC water for 25 min. Naked siRNA (lane 2, 5) and SiNP-DOX/siRNA nanocomposites (Lane 3, 6) are incubated with RPMI-1640 medium for 24 and 48 h. SiNP-DOX/siRNA nanocomposites (Lane 4, 7) are incubated with RPMI-1640 medium for 24 and 48 h followed by 1% heparin treatment. (Color figure online)

Fluorescent (PLQY ~ 25%) and water-dispersible SiNPs were synthesized as described in the Experimental Section and as we previously detailed [29]. DOX and siRNA were then sequentially loaded onto SiNPs via hydrophobic and electrostatic interactions, respectively, as shown in Fig. 1a. Before the addition of siRNA, the fabricated SiNP-DOX nanocomposites [28] were washed several times to remove free DOX. Washing was done until the fluorescence of the filtrate was undetectable (Fig. S1). To examine the formation of SiNP-DOX/siRNA nanocomposites, TEM, DLS, and agarose gel electrophoresis were carried out. TEM images revealed the spherical shape of the SiNP-DOX/siRNA nanocomposites, with a diameter of ~ 6.3 nm (Fig. 1b, c). The diameter of SiNPs and SiNP-DOX nanocomposites was ~ 2.7 and 4.2 nm, respectively (Fig. S2a, b). The hydrodynamic diameter of SiNP-DOX/siRNA nanocomposites as determined by DLS was ~ 7.2 nm, which was also obviously larger than those of pure SiNPs (~ 3.6 nm) and SiNP-DOX conjugates (~ 4.7 nm) (Fig. 1d), providing evidence of the loading of siRNA onto SiNP-DOX. It is worth noting that the successful binding of siRNA had very little influence on the optical properties of SiNP-DOX conjugates. As shown in Figs. 1e and S3, the UV–Vis–NIR absorption and PL spectra of SiNP-DOX conjugates displayed no obvious changes after siRNA loading. To confirm the siRNA binding capabilities of SiNP-DOX, agarose gel electrophoresis was performed after mixing the SiNP-DOX nanocomposite with siRNA at different SiNPs/siRNA (w/w) ratios. As shown in Fig. 1f, the migration of siRNA in the gel was gradually retarded with increasing ratios of SiNP-DOX. Almost no free siRNA could be detected at a w/w ratio above 150, demonstrating the complete binding of siRNA by SiNP-DOX conjugates. The loading efficiency of siRNA quantitatively calculated by Image J was as high as ~ 98% at the w/w ratio of 150 (Fig. S4).

The capability of SiNP-DOX/siRNA to protect siRNA from nuclease degradation is evaluated by incubating SiNP-DOX/siRNA with culture medium (RPMI-1640) or RNase A. As depicted in Figs. 1g and S5, the band intensity of naked siRNA decreased rapidly with time; only ~ 37% and 19% of the siRNA maintained its integrity after incubation with medium for 24 and 48 h, respectively (Fig. 1g, lane 2 and 5). In sharp contrast, ~ 100% siRNA loaded onto SiNP-DOX/siRNA nanocomposites was released by heparin, even after a 48-h incubation with medium (Fig. 1g, lane 7), suggesting that the loaded siRNAs were effectively protected from nuclease degradation. This protection could be attributed to steric hindrance on surfaces, consistent with several previous studies [15, 32]. Additionally, following the incubation of the SiNP-DOX/siRNA nanocomposites with RNase A for 1 h, the loaded siRNA was hardly degraded, consistent with its protection by the SiNP-DOX/siRNA nanocomposites (Fig. S6). Moreover, the SiNP-DOX/siRNA nanocomposites had extremely high storage or photostability (Fig. S7). The fluorescence intensity remained stable in different incubation conditions (i.e., PBS and RPMI-1640 medium) during 1 week at 37 °C. These results suggested the potential feasibility for the long-term analysis of the intracellular behavior of SiNPs-based nanocarriers via tracking of their fluorescence signals.

### Dual-Controlled Release of siRNA and DOX

**Fig. 2:**
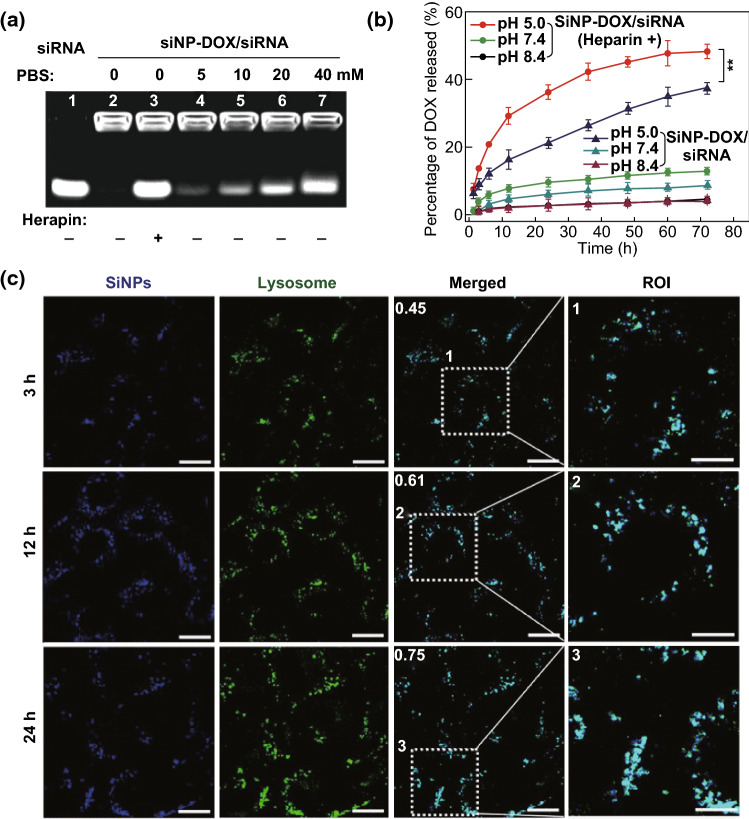
Dual-controlled release of siRNA and DOX from SiNP-DOX/siRNA nanocomposites and subcellular localization of SiNP-DOX/siRNA nanocomposites. **a** siRNA release in phosphate-buffered saline (PBS), lane 1: siRNA; lane 2: SiNP-DOX/siRNA nanocomposites; lane 3: SiNP-DOX/siRNA nanocomposites treated with 1% heparin; lane 4–7: SiNP-DOX/siRNA nanocomposites treated with different concentrations of PBS. **b** DOX release from SiNP-DOX/siRNA nanocomposites without or with heparin treatment (heparin +) at different pH values for different interval times. Data are presented as mean ± SD (*n* = 3). (**) *P* < 0.01, compared to those of the SiNP-DOX/siRNA nanocomposites group. **c** Confocal images of SiNP-DOX/siRNA nanocomposites (DOX = 5 μg mL^−1^, A_SiNPs_ = 1, siRNA = 100 nM) incubated with MCF-7/ADR cells for 3, 12, and 24 h. Scale bar = 25 μm. Pearson’s correlation coefficient (*R*_r_) values are displayed in white. (1, 2 and 3) Enlargement images of region of interests for MCF-7/ADR cells. Scale bar = 10 μm. The fluorescence of SiNPs and lysosomes is indicated as blue and green, respectively. (Color figure online)

Besides sufficient protection of siRNA from nuclease degradation, the controlled and efficient release of siRNA to the cytoplasm is required for successful siRNA delivery [10]. The siRNA loaded onto SiNP-DOX/siRNA nanocomposites could be completely released by 1% heparin (Fig. 2a, lane 3). To further model the intracellular environment, PBS containing different concentrations of the phosphate group was used to promote the release of siRNA from the SiNP-DOX/siRNA nanocomposites. As obviously apparent in Figs. 2a and S8, siRNA was gradually released from the nanocomposites as the phosphate group concentration increased. After incubation with 10 mM PBS for 1 h, more than 45% of the siRNA was released. When the concentration of PBS increased to 40 mM, the release of siRNA was ~ 82%. It is reasonable to expect that the siRNA can be dissociated from the SiNP-DOX/siRNA nanocomposites after their cellular internalization into endosomes as endosomes have a much higher concentration of endogenous phosphate ions than the surrounding environment [15, 33]. Furthermore, the DOX release behavior was quantitatively studied in an acidic-to-basic environment. A pH-dependent release of DOX was evident, i.e., more DOX molecules were released in pH 5.0 than those released at pH 7.4 and 8.4 (Fig. 2b, line graphs with triangular dots), in accordance with the release behavior of SiNP-DOX (Fig. S9). Typically, after a 72-h incubation at pH 8.4 and 7.4, < 4% and 9% of the loaded DOX was released from SiNP-DOX/siRNA nanocomposites, respectively. In sharp contrast, ~ 37% of the loaded DOX was released from the SiNP-DOX/siRNA nanocomposites upon incubation in an acidic environment (pH 5.0). It can be speculated that the SiNP-DOX/siRNA nanocomposites maintain their integrity in cell culture or blood before reaching the action site (e.g., endosomes or lysosomes). Notably, due to the presence of siRNA, the release of DOX from SiNP-DOX/siRNA was slower than from SiNP-DOX. When the SiNP-DOX/siRNA nanocomposites were pretreated with 1% heparin (i.e., siRNA dissociated), the increased release of DOX molecules was obvious, especially at pH 5.0 (Fig. 2b, red line graphs with circular dots). For example, ~ 48% of the loaded DOX was released from SiNP-DOX/siRNA nanocomposites—termed SiNP-DOX/siRNA (Heparin +)—after a 72-h incubation at pH 5.0, which was almost equal to SiNP-DOX nanocomposites (i.e., ~ 50%, Fig. S9).

### Intracellular Trafficking

To further demonstrate the controlled release behavior of SiNP-DOX/siRNA nanocomposites inside cells, we first investigated their subcellular localization. LysoTracker Green DND-26 was used to label lysosomes. After a 3-h incubation, the fluorescence signals of SiNPs were clearly observed as blue fluorescent dots (Fig. 2c, left panel). As shown in the merged confocal microscopic images, cyan dots were distinctly found, indicating the colocalization of SiNPs (blue signals) with lysosomes (green signals). The value of the Pearson’s correlation coefficient (*R*_*r*_, one standard measure for analyzing colocalization) was calculated as 0.45, providing a quantitative confirmation of the good colocalization between SiNPs and lysosomes. At incubation times of 12 and 24 h, the *R*_*r*_ values increase to 0.61 and 0.75, respectively. These results clearly demonstrated that the co-delivered SiNPs-based nanocomposites (i.e., SiNP-DOX/siRNA) can be retained in lysosomes after cellular internalization. DOX and siRNA may be released from nanocomposites responsive to acidic (pH 5.0–5.5) and phosphate-enriched environment of lysosomes [15, 33, 34].

**Fig. 3:**
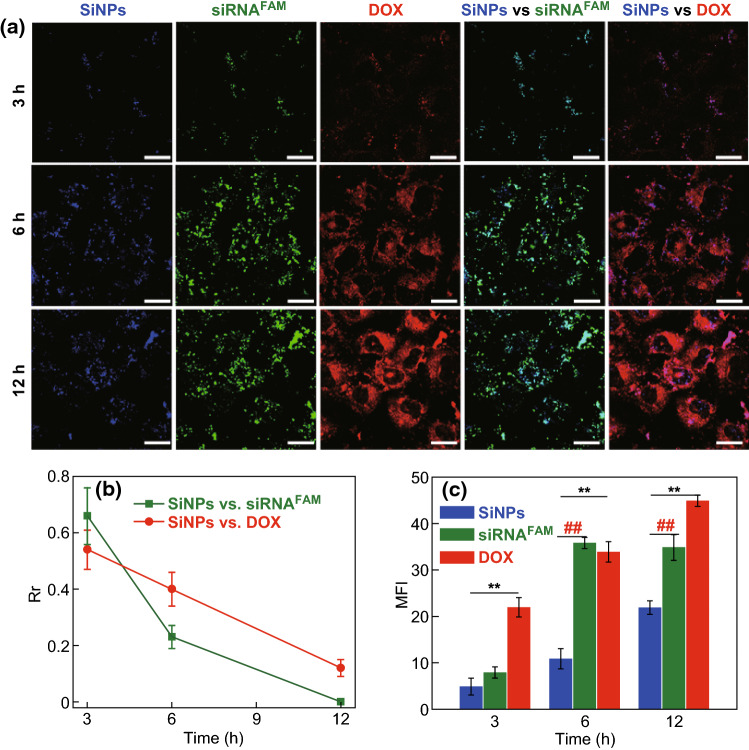
Intracellular trafficking of SiNP-DOX/siRNA nanocomposites (SiNP-DOX/siRNA^FAM^, siRNA labeled with FAM) in MCF-7/ADR cells. The fluorescence of SiNPs, siRNA^FAM^, and DOX is indicated as blue, green, and red, respectively. **a** Confocal images of localization of SiNP-DOX/siRNA^FAM^ (DOX = 5 μg mL^−1^, A_SiNPs_ = 1, siRNA^FAM^ = 100 nM) at different time points. Scale bar = 25 μm. **b** Summarized *R*_r_ values as a function of time. **c** The geometric mean fluorescence intensity (MFI) of SiNPs, siRNA^FAM^ and DOX in a calculated via Leica LAS AF Lite software. (**) *P* < 0.01, compared to the DOX group. (##) *P* < 0.01, compared to the siRNA^FAM^ group. Data represent as mean ± SD (*n* = 3)

The intracellular dissociation of siRNA and DOX from SiNPs-DOX/siRNA nanocomposites was followed visually, taking advantage of the strong and stable fluorescence of SiNPs (Fig. 3). siRNA was labeled with the organic dye FAM (designated siRNA^FAM^). After a 3-h incubation, both the fluorescence signals of siRNA^FAM^ (green) and DOX (red) were well colocalized with that of SiNPs (blue), and the corresponding *R*_*r*_ values were 0.66 (SiNPs vs siRNA^FAM^) and 0.54 (SiNPs vs DOX) (Fig. 3a, b). The results suggested that siRNA and DOX molecules were still loaded on SiNPs-based nanocarriers at 3 h. At 6 h, an obvious dissociation of siRNA was observed, as demonstrated by the separation of green (siRNA^FAM^) fluorescence from blue (SiNPs). Accordingly, the *R*_*r*_ value decreased to 0.23 between fluorescent channels of siRNA^FAM^ (green) and SiNPs (blue). When the incubation time was extended to 12 h, the *R*_*r*_ value for siRNA^FAM^ and SiNPs became close to 0, suggesting a more extensive release of siRNA. For DOX, the *R*_*r*_ value of red (DOX) and blue fluorescence (SiNPs) gradually decreased to 0.40 and 0.12 at 6 and 12 h incubation, respectively. The relatively rapid release of siRNA (~ 12 h) would be beneficial for intracellular DOX accumulation, as evidenced by the enhanced mean fluorescence intensity of DOX released from the SiNPs (Fig. 3c), because the time would be sufficient time for siRNA-mediated MDR1 gene silencing [35].

### In Vitro Gene-Silencing Efficiency

**Fig. 4:**
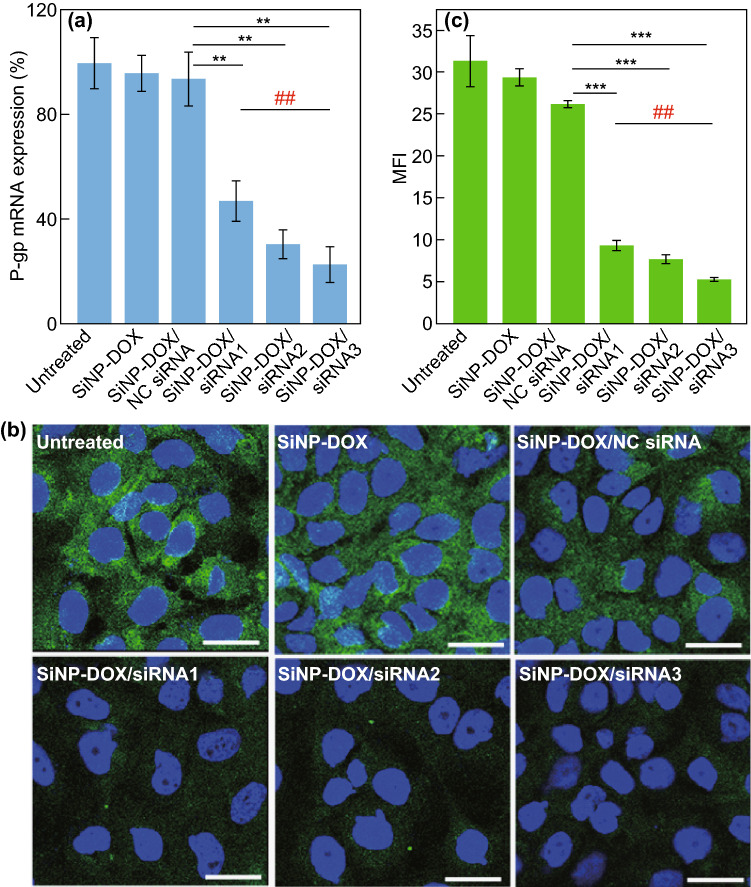
Gene-silencing efficiency in vitro. **a** Suppression of MDR1 mRNA level quantified by qRT-PCR. (**) *P* < 0.01, compared to the SiNP-DOX/NC siRNA group. (##) *P* < 0.01, compared to the SiNP-DOX/siRNA1 group. **b** The expression of P-gp evaluated by immunofluorescence staining. Scare bar = 25 μm. The fluorescence of P-gp and nucleus is indicated as green and blue, respectively. **c** The geometric mean fluorescence intensity of P-gp in **b** calculated using LAS AF Lite software. (***) *P* < 0.001, compared to the SiNP-DOX/NC siRNA group. (##) *P* < 0.01, compared to the SiNP-DOX/siRNA1 group. Data represent as mean ± SD (*n* = 3)

The silencing efficiency of SiNP-DOX/siRNA nanocomposites for the MDR1 gene was further evaluated at the mRNA and protein levels. It is worth noting that we chose three types of siRNA strands with different G+C contents (i.e., siRNA1, ~ 29%; siRNA2, ~ 38%; siRNA3, ~ 43%) targeting the MDR1 gene to prepare the SiNP-DOX/siRNA1, SiNP-DOX/siRNA2, and SiNP-DOX/siRNA3 nanocomposites. As shown in Fig. 4a, qRT-PCR analysis demonstrated that the transcription of MDR1 gene was preserved by > 90% upon treatment with the SiNP-DOX and SiNP-DOX/NC siRNA nanocomposites, suggesting the marginal influence of pure SiNPs, DOX, or NC siRNA on P-gp mRNA expression. In contrast, SiNP-DOX/siRNA nanocomposites induced significant knockdown of P-gp mRNA expression in MCF-7/ADR cells. The silencing efficiency of SiNP-DOX/siRNA nanocomposites ranged from ~ 53% (SiNP-DOX/siRNA1) to ~ 80% (SiNP-DOX/siRNA3), with an increase of 1.5-fold as the G+C content of siRNA increased from 29% (siRNA1) to 43% (siRNA3). The relatively higher G+C content may stabilize siRNA duplexes and increase the binding affinity for target mRNA [36]. The down-regulation of P-gp mRNA expression induced by SiNP-DOX/siRNA nanocomposites in MCF-7/ADR cells was also confirmed by agarose gel electrophoresis (Fig. S10). In addition, to examine the silencing effect of SiNP-DOX/siRNA nanocomposites at the protein level, immunofluorescence staining was performed to directly visualize P-gp protein expression (Fig. 4b). It was clearly evident that untreated MCF-7/ADR cells displayed high fluorescence intensity (green), suggesting the overexpression of P-gp protein. After treatment with SiNP-DOX or SiNP-DOX/NC siRNA nanocomposites, the fluorescence intensity of the cells decreased very little compared to that of untreated MCF-7/ADR cells. In sharp contrast, very weak green fluorescence signals were observed in the SiNP-DOX/siRNA nanocomposites-treated groups, indicating the extremely low expression of P-gp protein. Quantitative determination of the geometric mean fluorescence intensity with CLSM software is shown in Fig. 4c, which corroborated the G+C content-dependent silencing efficiency of SiNP-DOX/siRNA nanocomposites on P-gp protein expression.

### Reversal of Drug Resistance of MCF-7/ADR Cells

**Fig. 5:**
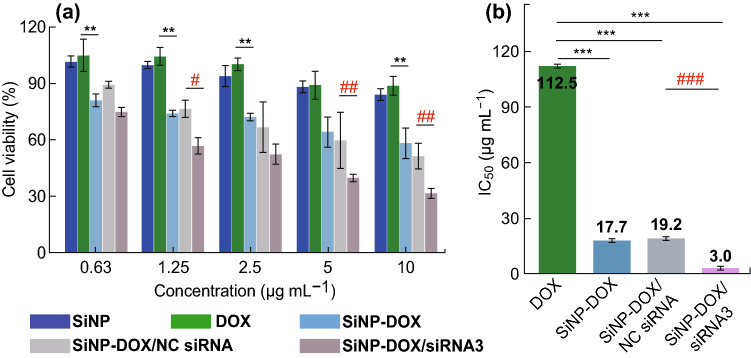
In vitro cytotoxicity assay. **a** After the 72-h incubation, the MCF-7/ADR cell viabilities of free SiNPs (A_SiNPs_: 0.125–2), free DOX (0.63–10 μg mL^−1^), SiNP-DOX conjugates (A_SiNPs_: 0.125–2; DOX: 0.63–10 μg mL^−1^), SiNP-DOX/NC siRNA and SiNP-DOX/siRNA nanocomposites (A_SiNPs_: 0.125–2; DOX: 0.63–10 μg mL^−1^; siRNA: 12.5–200 nM) were determined using the MTT assay. **b** IC_50_ values of different kinds of agents calculated from the cell viability assays. Data represent as mean ± SD (*n* = 4), (**) *P* < 0.01, (***) *P* < 0.001, compared to the free DOX group; (#) *P* < 0.05, (##) *P* < 0.01, (###) *P* < 0.001, compared to the SiNP-DOX-NC group

To examine whether the efficient knockdown of MDR1 gene mediated by SiNP-DOX/siRNA could induce distinct enhancement of chemotherapeutic efficiency, the cytotoxicity of SiNPs, DOX, SiNP-DOX, SiNP-DOX/NC siRNA, and SiNP-DOX/siRNA nanocomposites on MCF-7/ADR cells was assessed using the MTT assay (Fig. 5a). Viability was preserved in cells treated with free DOX (0.63–10 μg mL^−1^), with ~ 90% viability even after the 72-h incubation. Due to the overexpression of P-gp protein, a very low accumulation of the therapeutic agent (DOX) was evident in free drug-treated MCF-7/ADR cells compared to that in MCF-7 cancer cells (Fig. S11). Notably, SiNPs-treated cells also maintained approximately 90% viability, suggesting that SiNPs had little toxic effect on MCF-7/ARD cells. Interestingly, the cell viability was reduced to ~ 60% when MCF-7/ADR cells were incubated with the maximum concentration of SiNP-DOX (A_SiNPs_: 2; DOX: 10 μg mL^−1^) for 72 h. The result is reasonable because nanomaterial-based carriers have the ability to circumvent drug resistance mechanisms to a certain extent [11–19]. Comparatively, when MCF-7/ADR cells were treated with a corresponding concentration of SiNP-DOX/siRNA3 nanocomposites (A_SiNPs_: 2; DOX: 10 μg mL^−1^; siRNA: 200 nM), the cell viability decreased to 30%. Similar results were observed in the other two SiNP-DOX/siRNA nanocomposites-treated groups (Fig. S12), while the viability of SiNP-DOX/NC siRNA-treated cells remained at ~ 50% at this concentration (A_SiNPs_: 2; DOX: 10 μg mL^−1^; siRNA: 200 nM). The IC_50_ was further analyzed based on the results of MTT assay (Fig. 5a; Figs. S13 and S14). As shown in Fig. 5b, the IC_50_ values for free DOX, SiNP-DOX, SiNP-DOX/NC siRNA, and SiNP-DOX/siRNA3 nanocomposites were calculated as 112.5, 17.7, 19.2, and 3.0 μg mL^−1^, respectively. Of particular note, for SiNP-DOX/siRNA3, there was a 36.5-fold decrease in IC_50_ in comparison with that of free DOX, indicating the high therapeutic efficiency of the resultant nanocomposites for drug-resistant breast cancer cells (MCF-7/ADR). These data clearly demonstrated that co-delivery of siRNA and DOX by SiNPs-based nanocarriers can effectively reverse MDR by inhibiting P-gp expression and can coincidentally increase the drug sensitivity of MCF-7/ADR cells, resulting in the highly efficient killing of MDR cancer cells.

## Conclusions

We developed a novel fluorescent SiNPs-based nanomedicine platform, which is useful for imaging-guided co-delivery of siRNA and doxorubicin, enabling the enhancement of therapeutic efficacy in drug-resistant cancer cells. The nanomedicine platform (SiNP-DOX/siRNA nanocomposites) displayed dual-controlled release of siRNA and DOX molecules, which could be analyzed for prolonged periods in live cells by tracking fluorescence signals of SiNPs. The disassociated siRNA from SiNP-DOX/siRNA nanocomposite obviously down-regulated the expression of P-gp at the mRNA and protein levels (~ 80%), thus ensuring the sustained retention of released DOX in MCF-7/ADR cells. The SiNP-DOX/siRNA nanocomposites potently induced MCF-7/ADR cell death, as evident from the 36.5-fold decrease in IC_50_ (3 μg mL^−1^) compared to that of the free DOX group (112 μg mL^−1^), which overcame drug resistance. The results demonstrate the effectiveness of fluorescent SiNPs for the imaging-guided co-delivery of siRNA and DOX for therapy of MCF-7/ADR cells, and pave the way for the development of nanomedicines for MDR cancer cells. Of note, although silicon is distinguished by its low- or non-toxicity, numerous pioneering studies conducted by Professor Leong have revealed that nanoparticles may induce endothelial leakiness [37–40]. Therefore, further understanding of the behavior of SiNPs and SiNP-DOX/siRNA nanocomposites in vivo requires further investigation for their potential clinical application.

## Electronic Supplementary Material

Below is the link to the electronic supplementary material.

Supplementary material 1 (PDF 1017 kb)
