# Supplementary material for: Photostable and Biocompatible Fluorescent Silicon Nanoparticles for Imaging-Guided Co-Delivery of siRNA and Doxorubicin to Drug-Resistant Cancer Cells
Source: Nanomicro Lett. 2019 Mar 25;11:27. doi: 10.1007/s40820-019-0257-1 (PMC7770907; doi:10.1007/s40820-019-0257-1)
Supplement: Supplementary file 1 — Supplementary material 1 (PDF 1017 kb) [file 40820_2019_257_MOESM1_ESM.pdf]

Supporting Information for

Photostable and Biocompatible Fluorescent Silicon Nanoparticles for Imaging-Guided Co-Delivery of siRNA and Doxorubicin to Drug-Resistant Cancer Cells

Daoxia Guo[1, #], Xiaoyuan Ji[1, #], Fei Peng[1], Yiling Zhong[1], Binbin Chu[1], Yuanyuan Su[1,*], Yao He[1,*]

[1]Laboratory of Nanoscale Biochemical Analysis, Institute of Functional Nano and Soft Materials (FUNSOM), Jiangsu Key Laboratory for Carbon-Based Functional Materials and Devices, Soochow University, Jiangsu, Suzhou 215123, People's Republic of China

[#]Daoxia Guo and Xiaoyuan Ji contributed equally to this work

*Corresponding authors. E-mail: suyuanyuan@suda.edu.cn (Yuanyuan Su); yaohe@suda.edu.cn (Yao He)

Supplementary Figures and Discussion

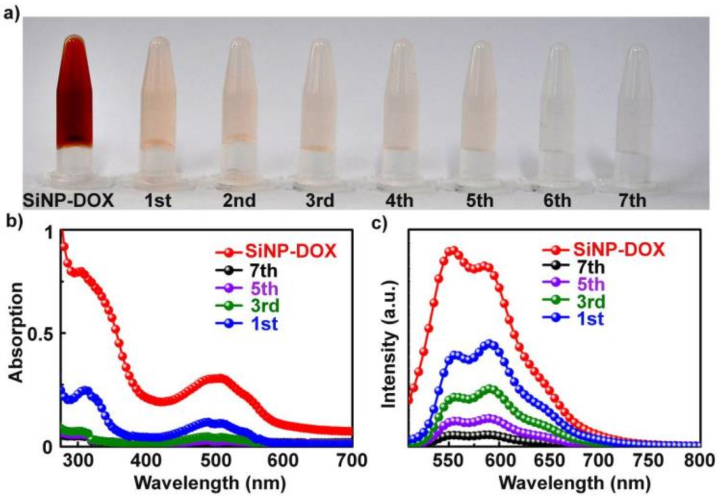

Fig. S1 Purification of SiNP-DOX conjugates. **a** Photos of the resultant SiNP-DOX conjugates and filtrate obtained at different wash times using 10 kDa Nanosep centrifugal devices through centrifugation (6000 rpm/15 min, per time). **b** Photoluminescence and **c** absorption spectra of the SiNP-DOX conjugates and filtrates obtained at the first, third, fifth and seventh wash time

As shown in Fig. S1a, the filtrate gradually becomes transparent after seven times of ultrafiltration, indicating the successful removal of DOX. The result is further verified by photoluminescence (Fig. S1b) and absorption spectra (Fig. S1c) of the SiNP-DOX conjugates and filtrates.

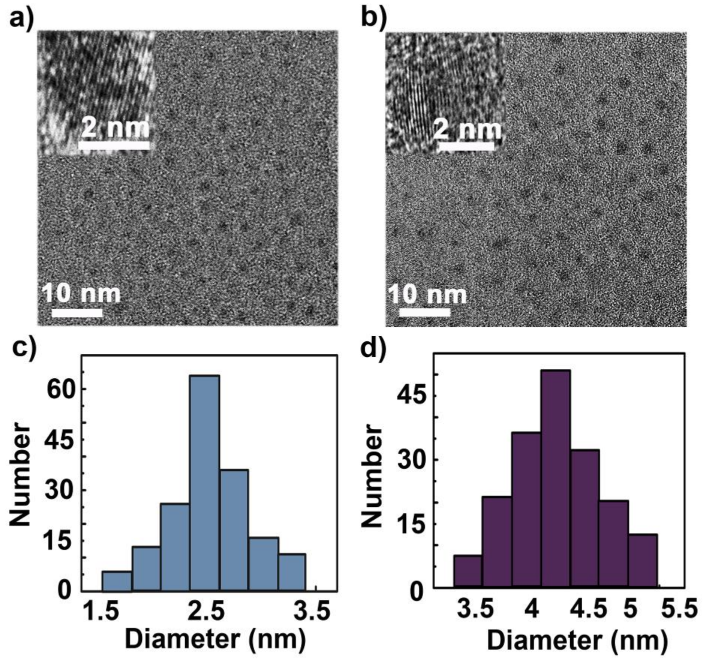

Fig. S2 a, b TEM images and **c, d** TEM diameter distributions of the as-prepared SiNPs and SiNP-DOX conjugates. Insets present HRTEM images

Figures S2a, b display TEM and HRTEM images of SiNPs and SiNP-DOX conjugates, in which SiNPs and SiNP-DOX conjugates appear as spherical particles with good monodispersity and excellent crystallinity. The size distributions of SiNPs and SiNP-DOX conjugates in Fig. S2c, d, calculated by measuring more than 200 particles, show average diameters of ~2.7 and ~4.2 nm, respectively. These data suggest that DOX molecules are successfully loaded onto SiNPs.

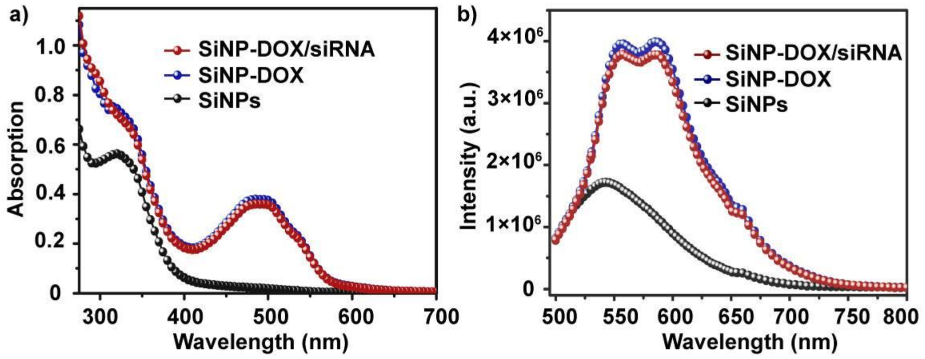

Fig. S3 a UV-Vis-NIR absorption and **b** photoluminescence spectra under excitation at 488 nm of pure SiNPs, SiNP-DOX conjugates, and SiNP-DOX/siRNA

The UV-Vis-NIR absorption of SiNP-DOX conjugates have no obvious change after siRNA loading, appearing characteristic absorption peaks of SiNPs and DOX molecules at ~320 and ~488 nm respectively. Similarly, the PL spectra of SiNP-DOX and SiNP-DOX/siRNA composites both preserve the characteristic PL peaks of DOX at λ_{max} of ≈600 nm excited by 488 nm. Therefore, the successful binding of siRNAs has no obvious influence on the optical properties of SiNP-DOX conjugates.

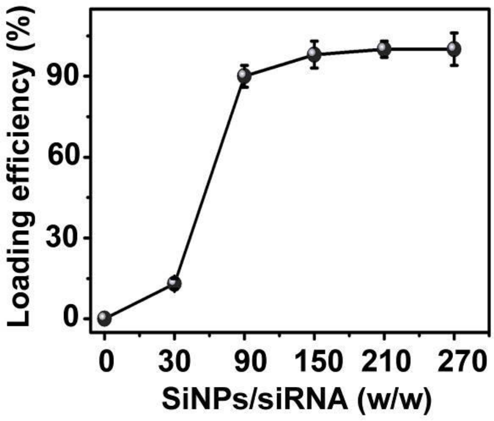

Fig. S4 Quantitative analysis by Image J software of the loading efficiency of siRNA trapped onto SiNP-DOX prepared at different SiNPs/siRNA (w/w) ratios. Data represent as mean ± SD (n = 3)

As analyzed by Image J software, the migration of siRNA in the gel is gradually retarded with increasing ratios in SiNP-DOX conjugates. The loading efficiencies of siRNA quantitatively calculated by Image J are ~13%, 90%, ~ 98%, ~100%, and ~100% at different SiNPs/siRNA (w/w) ratios (i.e., 30, 90, 150, 210, and 270).

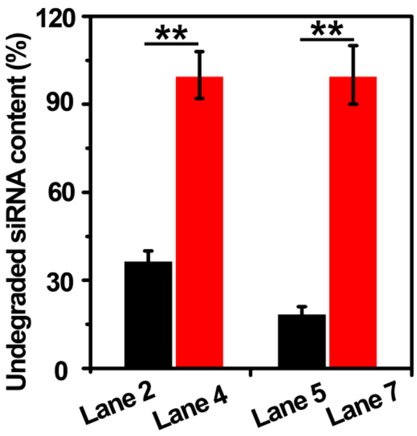

Fig. S5 Quantitative analysis of Fig. 1g by Image J software of the band intensity of siRNA and SiNP-DOX/siRNA nanocomposites incubated in culture medium (RPMI-1640) for 24 (lane 2 and 4) and 48 h (lane 5 and 7). Data represent as mean ± SD (n = 3), (**) $p < 0.01$, compared to free siRNA group

The band intensity of naked siRNAs weakens rapidly with time; only ~ 37% or 19% of the siRNAs maintains its integrity after incubation with medium for 24 or 48 h (lane 2 and 5). On the contrary, approximately 100% siRNAs loaded onto SiNP-DOX/siRNA nanocomposites can be released by heparin after 24 and 48-h incubation with medium (lane 4 and 7).

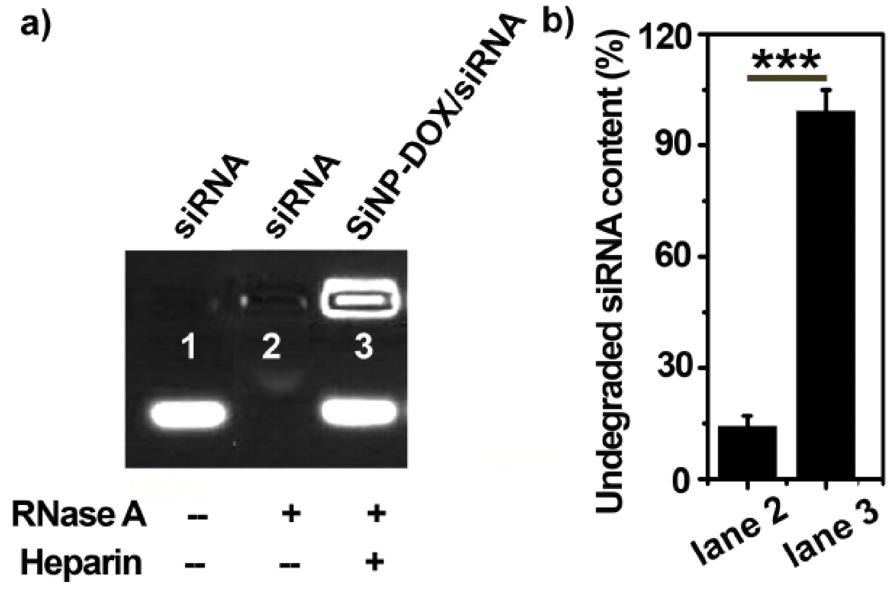

Fig. S6 RNase A protection assay. **a** RNase A protection assay by agarose gel electrophoresis. Lane 1: naked siRNA; lane 2: naked siRNA treated with RNase A; lane 3: SiNP-DOX/siRNA nanocomposites (SiNPs/siRNA (w/w) ratio of 210) treated with RNase A. **b** Quantitative analysis of Fig. S6a with Image J software, while the band intensity of lane 1 is taken as 100%. (***) P<0.001. Data represent as mean ± SD (n = 3)

It can be found that naked siRNAs is rapidly degraded by RNase A, where only ~15% siRNA keeps its integrity. Of particular note, ~100% siRNAs loaded onto SiNP-DOX/siRNA nanocomposites maintain intact after RNase A treatment, suggesting that the loaded siRNAs are effectively protected from nuclease degradation.

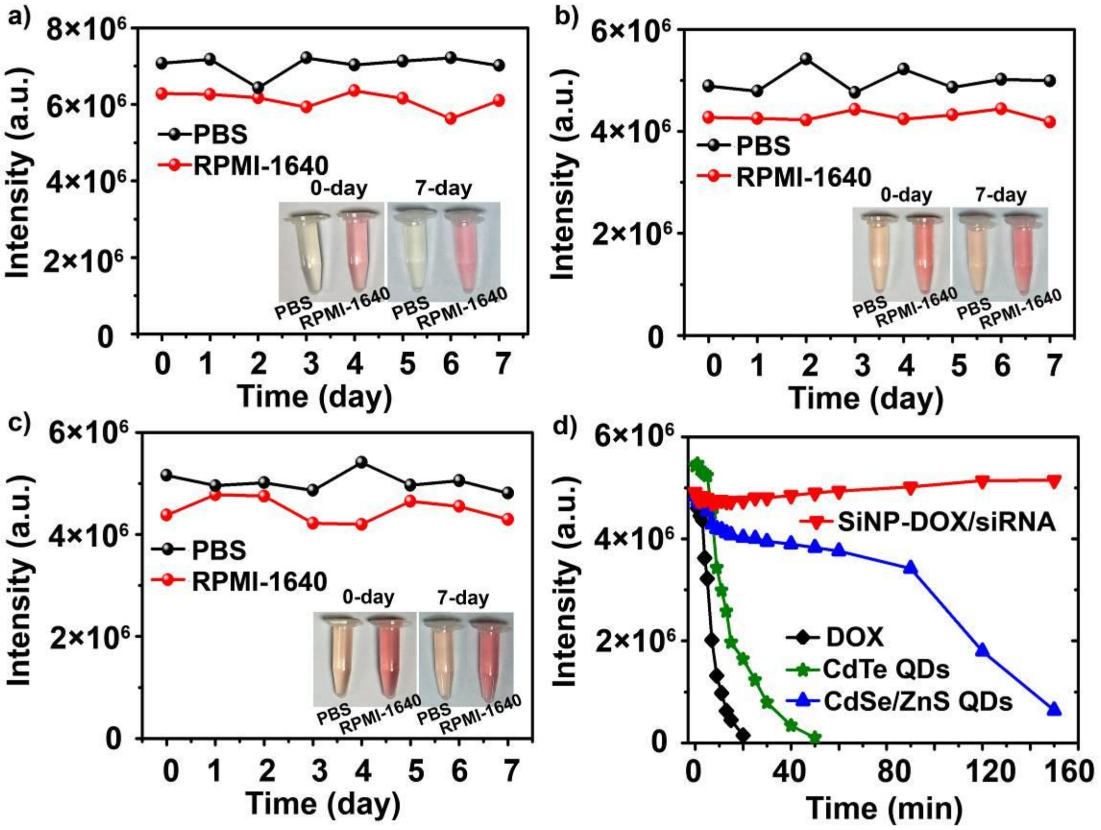

Fig. S7 Storage-/photo-stability investigation of SiNP-DOX/siRNA nanocomposites. The fluorescent intensity of **a** SiNPs, **b** SiNP-DOX, and **c** SiNP-DOX/siRNA nanocomposites in PBS and RPMI-1640 medium during seven days at 37 °C. Insets present digital photos of PBS or medium solutions containing SiNPs, SiNP-DOX and SiNP-DOX/siRNA nanocomposites at zero and seven day under ambient. **d** Photostability comparison of DOX, CdTe, and CdTe/ZnS quantum dots, and SiNP-DOX/siRNA under continuous UV irradiation (365 nm, 450 W xenon lamp)

SiNPs, SiNP-DOX conjugates, and SiNP-DOX/siRNA nanocomposites possess excellent storage stability in different incubation conditions (i.e., PBS and RPMI-1640 medium) during one week at 37 °C. As verified by the insets, the prepared SiNPs, SiNP-DOX conjugates and SiNP-DOX/siRNA nanocomposites are stable in ambient environment, appearing transparent aqueous solutions without obvious aggregation. Additionally, SiNP-DOX/siRNA nanocomposites have extremely high photostability (the fluorescence intensity remains almost unchanged during 150 min irradiation, while the fluorescence intensity of DOX and QDs is gradually quenched).

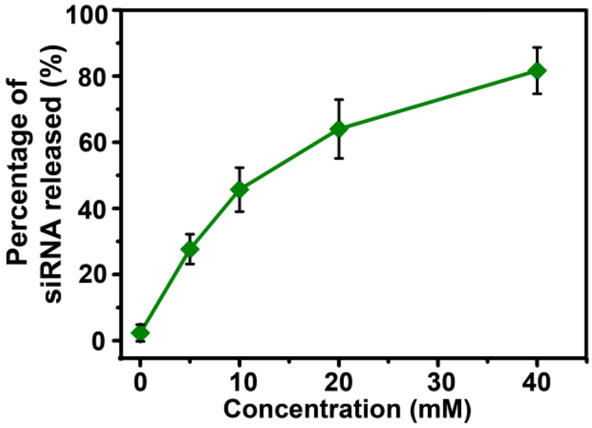

Fig. S8 Quantitative analysis of the band intensity of siRNA released from SiNP-DOX/siRNA nanocomposites treated with PBS (Fig. 2a). The band intensity of naked siRNA is taken as 100%. Data represent as mean ± SD (n = 3)

It is obviously found that the siRNAs are gradually dissociated from the nanocomposites as the phosphate group concentration increases. About 28%, 46%, 64%, and 82% of the loaded siRNA could be released from the nanocomposites when the concentration of phosphate increases to 5, 10, 20, and 40 mM, respectively.

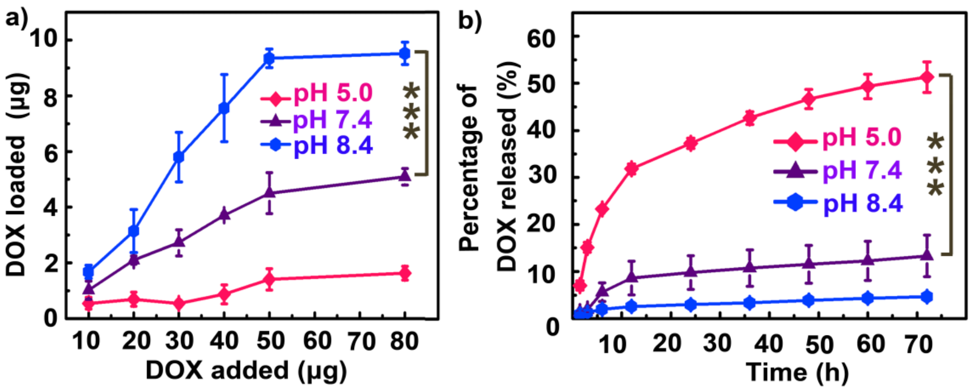

Fig. S9 a Loading and **b** releasing behavior of fluorescent SiNP-DOX conjugates under pH control (pH 5.0, 7.4 and 8.4) at 37 °C. (***) P<0.001, compared with pH 5.0 group. Data represent as mean ± SD (n = 3).

Typically, the DOX loading and releasing behavior is pH-dependent. Figure S9a shows that a maximal DOX absorption is uncovered ~10 μg when the weight of DOX added is larger than 50 μg under basic environment. As shown in Fig. S9b, DOX molecules loaded on SiNPs are stable in basic and neutral buffer (e.g., ~4% or ~11% DOX is released from SiNPs at pH 8.4 or 7.4 after 72-h incubation, respectively). In sharp contrast, a maximal amount of DOX released from SiNPs at pH 5.0 is ~50% during 72 h, which is due to the improved hydrophilicity and higher solubility of DOX in acidic environments.

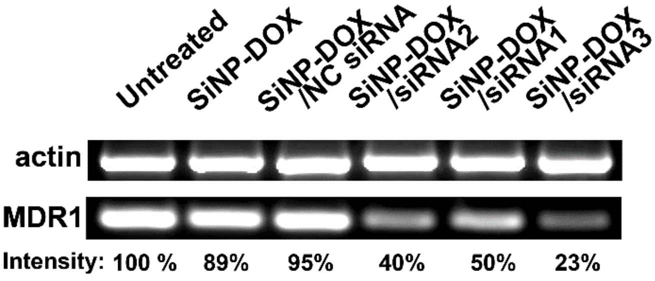

Fig. S10 Suppression of MDR1 mRNA level analyzed by agarose gel electrophoresis. The intensity is quantified with Image J software and the band intensity of untreated group is taken as 100%

The agarose gel electrophoresis result reflects that SiNP-DOX or SiNP-DOX/NC siRNA nanocomposites have little effect on the transcription of MDR1 gene. Notably, SiNP-DOX/siRNA nanocomposites could silence MDR1 mRNA expression in MCF-7/ADR cells with high efficacy (~50%, ~60%, and ~77% down-regulation for SiNP-DOX/siRNA1, SiNP-DOX/siRNA2 and SiNP-DOX/siRNA3 group, respectively).

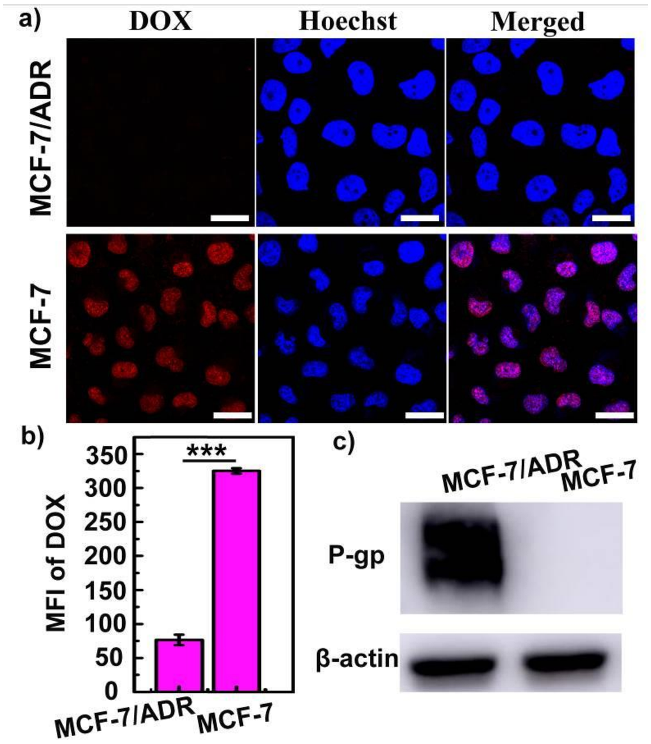

Fig. S11 Identification of DOX sensitivity in MCF-7/ADR and MCF-7 cells. **a** Confocal images of MCF-7/ADR and MCF-7 cells incubated with DOX (DOX=3 μg mL^{-1}) for 6 h. Scale bar = 25 μm. **b** Intracellular DOX uptake analyzed with flow cytometry in MCF-7/ADR and MCF-7 cells treated with free DOX (3 μg mL^{-1}) for 6 h. Data represent as mean ± SD (n = 3), (***) P<0.001. **c** Characterization of P-gp expression level in MCF-7/ADR and MCF-7 cells by immunoblotting

After 6-h incubation of DOX (3 μg mL^{-1}), feeble red signals are observed in the cytoplasm due to the poor retention of free DOX, which could be pumped out persistently by the P-gp efflux. In contrast, most of DOX fluorescence is distributed in nucleus in MCF-7 cells.

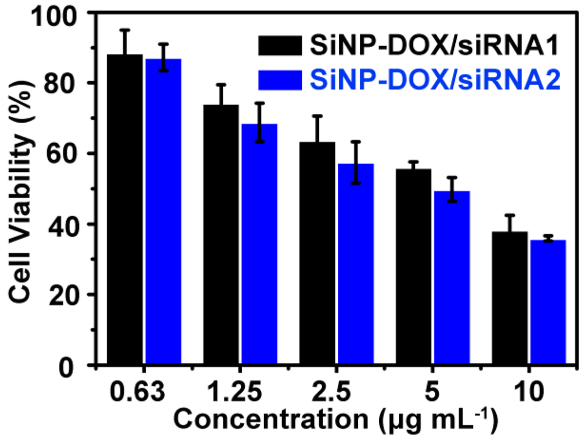

Fig. S12 Cytotoxicity of SiNP-DOX/siRNA nanocomposites (siRNA1 and siRNA2, A_{SiNPs}: 0.125-2, DOX: 0.63-10 μg mL^{-1}, siRNA: 12.5-200 nM) in MCF-7/ADR incubated for 72 h at 37 °C. Data represent as mean ± SD (n = 4)

After treated with a corresponding concentration of SiNPs-DOX/siRNA1 and SiNPs-DOX/siRNA2 nanocomposites (A_{SiNPs}: 2; DOX: 10 μg mL^{-1}; siRNA: 200 nM) for 72 h, the cell viability of MCF-7/ADR cells can drop to 39% and 35%, respectively.

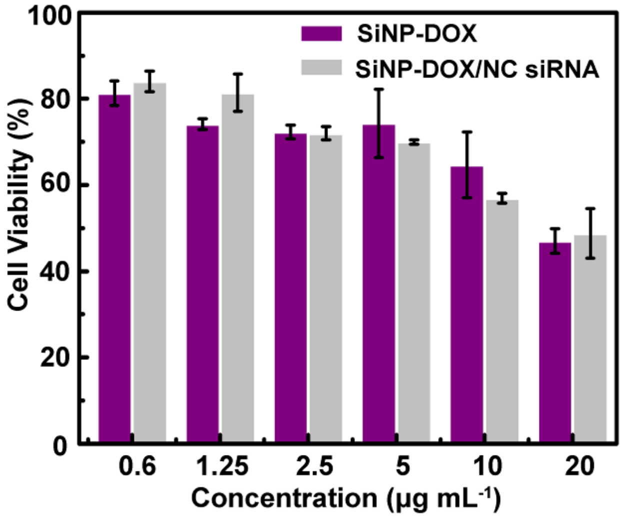

Fig. S13 *In vitro* concentration-dependent cytotoxicity of SiNP-DOX conjugates and SiNP-DOX/NC siRNA nanocomposites (A_{SiNPs}: 0.125-4, DOX: 0.63-20 μg mL^{-1}, siRNA: 12.5-400 nM) in MCF-7/ADR cells incubated for 72 h at 37 °C. Data represent as mean ± SD (n = 4)

After treated with a corresponding concentration of SiNPs-DOX and SiNPs-DOX/NC siRNA nanocomposites (A_{SiNPs}: 4; DOX: 20 μg mL^{-1}; siRNA: 400 nM), the cell viability of MCF-7/ADR cells can drop below 50%.

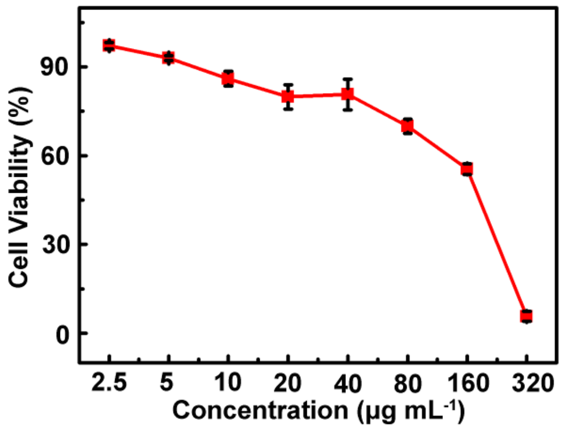

Fig. S14 *In vitro* concentration-dependent cytotoxicity of DOX (3.5-320 μg mL^{-1}) in MCF-7/ADR cells incubated for 72 h at 37 °C. Data represent as mean ± SD (n = 4)

After 72-h incubation of DOX (3.5-320 μg mL^{-1}), the cell viability of MCF-7/ADR cells gradually fell to ~12%.

Table S1 Sequences of three different types of P-gp siRNA and scramble siRNA

	**Sequences**
P-gp siRNA1 sense	5'-GGGAUAAAGAAAGCUAUUATT-3'
P-gp siRNA1 antisense	5'-GUGGGCACAAACCAGAUAATT-3'
P-gp siRNA2 sense	5'-GACCAGGUAUGCCUAUUAUTT-3'
P-gp siRNA2 antisense	5'-UAAUAGCUUUCUUUAUCCCTT-3'
P-gp siRNA3 sense	5'-AUAAUAGGCAUACCUGGUCTT-3'
P-gp siRNA3 antisense	5'-UUAUCUGGUUUGUGCCCACTT-3'
Scramble siRNA sense	5'-UUCUCCGAACGUGUCACGUTT-3'
Scramble siRNA antisense	5'-ACGUGACACGUUCGGAGAATT-3'

Table S2 Forward and reverse sequences of P-gp and β-actin primer

Primer	**Sequences**
P-gp F	5'-AGGAAGCCAATGCCTATGACTTTA-3'
P-gp R	5'-CAACTGGGCCCCTCTCTCTC-3'
β-actin F	5'-TCACCCACACTGTGCCCATCTACGA-3'
β-actin R	5'-CAGCGGAACCGCTCATTGCCAATGG-3'
